# Household food insecurity, income loss, and symptoms of psychological distress among adults following the Cyclone Amphan in coastal Bangladesh

**DOI:** 10.1371/journal.pone.0259098

**Published:** 2021-11-02

**Authors:** Ahmed Hossain, Bayes Ahmed, Taifur Rahman, Peter Sammonds, Shamrita Zaman, Shadly Benzadid, Md. Jakariya

**Affiliations:** 1 Department of Public Health, North South University, Dhaka, Bangladesh; 2 Global Health Institute, North South University (NSU), Bashundhara, Dhaka, Bangladesh; 3 Health Management BD Foundation, Sector 6, Uttara, Dhaka, Bangladesh; 4 Institute for Risk and Disaster Reduction (IRDR), University College London (UCL), London, United Kingdom; 5 Department of Disaster Science and Management, Faculty of Earth and Environmental Sciences, University of Dhaka, Dhaka, Bangladesh; University of Southern Queensland, AUSTRALIA

## Abstract

**Introduction:**

Cyclone Amphan swept into Bangladesh’s southwestern coast at the end of May 2020, wreaking havoc on food security and economic stability, as well as possibly worsening mental health. We studied the prevalence of post-cyclone stressors in adults following the cyclone and its association with symptoms of psychological distress.

**Methods:**

We conducted a cross-sectional study in a coastal district of Bangladesh. A five-item brief symptom rating scale was used to measure the symptoms of psychological distress. Household food insecurity was measured using the USAID Household Food Insecurity Access Scale questionnaire. We estimated adjusted prevalence ratios (aPRs) using robust log-linear models adjusted for potential confounders.

**Results:**

A total of 478 adults (mean [SD] age, 37.0[12.6] years; 169[35.4%] women) participated in the study. The prevalence of moderate-to-severe psychological symptoms and suicidal ideation was 55.7% and 10.9%, respectively. Following the cyclone, 40.8% of the adults reported severe food insecurity, and 66% of them reported moderate-to-severe mental health symptoms. Also, 54.4% of women and 33.7% of men reported severe food insecurity in the households. Moreover, 25.5% of respondents reported no income or a significant income loss after the cyclone, and 65.5% of them had moderate-to-severe psychological symptoms. Also, 13.8% of respondents reported housing displacement because of severely damaged houses, and 68.2% of them reported moderate-to-severe psychological symptoms. The high prevalence of mental health symptoms was found in women (aPR = 1.41, 95% CI = 1.06–1.82), people with severe food insecurity (aPR = 1.63, 95% CI = 1.01–2.64), and people who lost jobs or lost a major income source (aPR = 1.25, 95% CI = 1.02–1.54).

**Conclusion:**

Following cyclone Amphan, many low-income individuals saw their income drop drastically while others were unemployed and living with severe food insecurity. The result suggests gender inequalities in food-security after the cyclone. Immediate action is needed to ensure household food-security for reducing the burden of mental illness. Rising opportunities of paid-jobs and decreasing income-loss, especially for the poor people, can have a protective impact on psychological distress. However, due to the high prevalence of severe psychological symptoms, long-term mental health services are required among the population of coastal Bangladesh.

## Introduction

Bangladesh is often swept away by coastal cyclones. The 1991 cyclone was among the worst tropical cyclones on record in the history of Bangladesh [1]. The cyclone caused at least 138,866 deaths in the last week of April that year [1]. Cyclone Nargis in 2008 devastated the southern delta region of Myanmar and southeast of Bangladesh, with extreme storm-surge flooding [1, 2]. More than 130,000 people were killed due to cyclone Nargis, mostly in neighboring country Myanmar. Around 3500 people in Bangladesh were killed in Sidr, which hit the coastal belt in 2007 [1]. Just two years later, in 2009, another cyclone, Aila, killed 190 people in the country [1]. It was categorized as a "Severe Cyclonic Storm". In 2013, Mahasen, known as a "Cyclonic Storm," killed 18 people [1]. Cyclone Amphan was another worst Bay of Bengal storm since 1991 [3]. These bring negative consequences on agricultural production, food-security, and livelihood for the people who live in the coastal areas.

Cyclone Amphan swept into eastern India and southwestern coast of Bangladesh on May 20, 2020, evening with pounding winds and torrential rains, inflating sea waters causing as high as 12 feet tidal surges [3, 4]. According to various sources, over a million people were affected by Cyclone Amphan in Bangladesh’s nine coastal districts. In Bangladesh, some 10 million people were affected by Cyclone Amphan, killing at least 25 people [3]. In addition, half a million families might lose their homes [4]. The storm struck hard in the Satkhira district of Bangladesh. The high tides triggered by the Amphan cyclone destroyed the dam in four areas of Satkhira, flooding at least 20 villages [3]. It has also washed away some croplands and fish enclosures. The regions worst affected by cyclone Amphan were Shyamnagar, Ashashuni, and Kaliganj Upazila of Satkhira [4].

There have been growing numbers of studies concerning post-disaster mental distress, including post-traumatic stress disorder (PTSD), depression, anxiety, and suicidality [5–8]. A study reported an increased tendency of suicidal thoughts and suicidal attempts among the survivors within 12 months following a natural disaster [9]. A qualitative study investigated the population’s mental health needs and services in a cyclone-affected area in coastal Bangladesh [10].

It is challenging to understand psychological distress with suicide risk factors because genetic variables, biological and environmental variables, and individual coping behaviors contribute to the etiology of poor mental health [11, 12]. A Canadian study found a significant causal association between food insecurity and suicidal ideation [13]. Suicidal ideation (SI) in disaster-prone areas could be intensified due to food insecurity [14, 15]. Financial difficulties and job loss were identified in individuals [16]. Food insecurity, job loss or loss of income, and living conditions are the post-disaster stressors that could affect the psychological distress with SI among the disaster survivors. This study will be the first-ever of its kind to investigate the household food insecurity among adults following cyclone Amphan and its effects on psychological distress with SI.

The current study’s main objective was to investigate the relationship between post-cyclone stressors and symptoms of psychological distress in adults in the face of cyclone Amphan. Understanding these relationships can help mental health practitioners develop and execute programming efforts addressing vulnerable, high-risk populations of psychological disorders in post-cyclone settings.

## Materials and methods

### Study site

In August 2020, we conducted a cross-sectional survey among cyclone Amphan survivors (18 years or older) residing in the Satkhira district of Bangladesh. We randomly selected two Upazilas- Shyamnagar and Ashashuni from three affected Upazilas of Satkhira district. Cyclone Amphan had impacted over a million people in these two Upazilas- Shyamnagar and Ashashuni. A map of study regions and sample allocation is given in the **(S1 Table in S1 File)**.

### Sample size estimation

We aim to include prospective participants from the Satkhira district via a two-stage cluster sampling technique. First, the expected prevalence of moderate-to-severe mood disorder is considered as 50%. Second, we assumed an intra-cluster correlation for this disorder to be 0.02. Thus, we want to be 95% certain that our estimate of the prevalence of mood disorder is within 5% of the true population value, and we intend to sample 270 individuals per cluster. The estimation provides a total of 2 clusters that need to be sampled to meet this study’s specifications. The result of the sample size estimation using the epiR package of R is given in the (**S1 Appendix in S1 File)** [17]. The methodology used in this function follows the approach described by Bennett et al. (1991) [18].

### Data collection

We conducted a random sampling procedure to collect data from the two Upazilas on Bangladesh’s coastal side. In the two-stage sampling procedure, we selected first the two Upazilas from the five of affected Upazilas in Satkhira district. Next, we selected at least 270 households from each selected Upazilas with a target of 540 households in the study. We applied a systematic sampling technique for household selection, and the first household was randomly chosen from the approximate geographical center of the Upazila. Data collectors proceeded to the next closest household until 270 households were sampled. We had a single respondent per household interviewed by a semi-structured questionnaire, preferably the household’s head. The female head of the household or other available adult household members were surveyed when the male head of the household was not available. We ensured that the participant lived in the same house for at least a year. We excluded any psychiatric disorder patients, cancer patients, or pregnant mothers from the study. Patients with psychiatric disorders were ruled out by inquiring if they took any medicine for psychiatric symptoms or illegal drugs.

### Recruitment and training

The data is obtained and cleaned by a team of 4 enumerators from the Health Management BD Foundation (HMBD). The HMBD Foundation, a local NGO is working on the coastal side of Satkhira districts since the hit of the cyclone Amphan, chose the local community leaders (called a chairman) from each of the selected two Upazilas. The local leader from each of the Upazilas assisted the HMBD foundation in recruiting two local university students from each Upazila. A team of data collectors was then built, including two persons, i.e., one from Upazila and another person from the HMBD foundation. One research investigator arranged a one-day online training session about ethics and data collection for the data collection team. The team was also briefed about the study objectives, methodology, and questionnaire. The researchers also taught data collectors the techniques of report building and preserving neutrality and informed on ethical problems, privacy concerns, cultural awareness, and risk management for mental health. A pilot study was arranged for the two study teams and evaluated as a single unit following the training. The aim was to observe the capacity to comprehend the relevant techniques and trouble-some situations while interviewing. We made necessary corrections following the piloting. Afterwards, each trained team visited their designated Upazila to collect data.

### Sociodemographic factors

Socio-demographics assessed included age, gender, marital status, education, pre-, and post-Cyclone family income, household family members, number of children, and household facilities. Age was categorized as 18–24, 25–34, 35–44, 45–54, and 55 and older. We categorized family members as ≤4, 5–6, and >6 members in the household. Many of the cyclone Amphan survivors worked in agricultural fields, fishing, or work as day labor and thus belonged to the low socioeconomic group.

### Wealth index

We also calculated the household wealth score based on the household materials. Wealth score is a social economics indicator for measuring the living standard of households. It can also be used as a proxy for food access. The index is based on the household’s ownership of selected assets, such as televisions and bicycles, materials used for housing construction, and types of water access and sanitation facilities. We generated the wealth score using principal component analysis and placed individuals on a continuous scale-based on the scores of the first principal component. The first principal component explains the largest proportion of the total variance, and it is used as the wealth index to represent the household’s wealth. The score is then ranked, after which it is subdivided into five equal stratums called wealth quintiles. The lowest quintile (quintile 1) represents the bottom 20%, i.e., poorest, and the upper quintile are the wealthiest, i.e., top 20% of the population.

### Outcome measurement: Psychological symptoms from 5-item brief symptom rating scale (BSRS-5)

The BSRS-5 contains five psychological symptoms and is commonly used for psychological screening disorders with excellent validity and reliability [19, 20]. BSRS-5 can also be used as an initial screen tool for identifying suicidal ideation [19]. The BSRS-5 is a 5-item, self-administered questionnaire that is derived from the 50-item brief symptom rating scale, which measures anxiety (feeling tense or high-strung), depression (feeling depressed or in a low mood), hostility (feeling easily annoyed or irritated), interpersonal sensitivity (feeling inferior to others), and additional symptoms (having trouble falling asleep in the past week). Each item’s score ranges from 0 to 4 (0, not at all; 1, a little bit; 2, moderately; 3, quite a bit; and 4, extremely). A total score on the BSRS-5 above 14 may indicate a severe mood disorder that requires immediate clinical advice [18]. Scores between 10 and 14 may indicate moderate mood disorders that require professional advice, and those between 6 and 9 could indicate mild mood disorders that require talking with family and friends.

### Suicidal ideation from BSRS-5

BSRS-5 has been widely used as suicidal ideation (SI) screening questionnaire for psychiatric inpatients, general medical inpatients, and community residents with different cut-off scores [19, 20]. For example, according to the study by Lung and Lee 2008, the optimal cut-off points derived from the ROC curve for these groups with SI were 7 in inferiority, hostility, and insomnia for the community group, and 12 in inferiority, hostility, depression, and insomnia for the general medical group [21].

### Post-cyclone stressors

Exposures to post-cyclone stressors were assessed by income loss, housing conditions, and household food insecurity. Three categories labelled the income loss: not lost any income or had ≤25% loss, had 25–75% loss, and not had an employment or more than 75% loss of income following cyclone compared to pre-cyclone income. Besides, we categorized housing conditions due to cyclone in three categories: the house was not damaged, the house was partially damaged but liveable, and the displacement from the house as the house was damaged and needed renovation for living. Household food insecurity was surveyed by using a questionnaire of the USAID Household Food Insecurity Access Scale (HFIAS) for Measurement of Food Access [22].

### Household Food Insecurity Access Scale (HFIAS)

HFIAS is an adaptation of the approach used to estimate the prevalence of household food insecurity and detect changes in the household food insecurity (access) situation of a population [22]. The HFIAS consists of two types of related questions. The first question type is called an occurrence question. Nine occurrence questions ask whether a specific condition associated with the experience of food insecurity ever occurred during the previous four weeks (30 days). Each severity question is followed by a frequency-of-occurrence question, asking how often a reported condition happened in the last four weeks. The 9-item questionnaire was composed of 3 different food insecurity domains (access) with a recall period of four weeks (30 days)—one of the questions asked about anxiety and uncertainty about the household food supply. Three of the questions are asked about the insufficient quality that includes variety and preferences of the type of food. Finally, five of the questions are asked about inadequate food intake and its physical consequences.

We categorized households into four levels of household food insecurity (access): food secure, mild, moderately, and severely food insecure. A food-secure household experiences none of the food insecurity conditions or just experiences worry, but rarely. A mild food-insecure household worries about not having enough food sometimes or often, and/or is unable to eat preferred foods, and/or eats a more monotonous diet than desired and/or some foods considered undesirable, but only rarely. Nevertheless, it does not cut back on quantity nor experience any of the three most severe conditions (running out of food, going to bed hungry, or going a whole day and night without eating). A moderate food-insecure household sacrifices quality more frequently by eating a monotonous diet or undesirable foods sometimes or often, and/or has started to cut back on quantity by reducing the size of meals or number of meals, rarely or sometimes. However, it does not experience any of the three most severe conditions. A severe food-insecure household has graduated to cutting back on meal size or several meals often, and/or experiences any of the three most severe conditions (running out of food, going to bed hungry, or going a whole day and night without eating), even as infrequently as rarely. In other words, any household that experiences one of these three conditions even once in the last four weeks (30 days) is considered severely food insecure.

### Statistical analysis

The questionnaire and data are available at https://osf.io/snj6e/. Data were analysed using R 3.6.2. All categorical variables are presented as frequencies and percentages. We investigated a directed acyclic graph (DAG) to adjust confounders in the associations between exposures and symptoms of psychological distress. This graphical method depicts hypothesized causal relationships and deduces the statistical associations implied by these causal relationships. We selected psychological symptoms categorizing into two groups (moderate-to-severe mood disorder and no or mild mood disorders) within the last week as the dependent variable. The considered exposures were sociodemographic factors, post-cyclone stressors, and the presence of underlying medical conditions. This figure was constructed through DAGITTY (http://www.dagitty.net/dags.html#) and is given in **(S1 Fig in S1 File)**.

We estimated prevalence ratios (PRs) and their confidence intervals using a robust log-linear model after adjusting for potential confounders. Cameron and Trivedi (2009) proposed using robust standard errors for the parameter estimates to manage mild violation of the distribution assumption that the variance equals the mean [23]. We used the R package "sandwich" to obtain the robust standard errors (SEs) in a Poisson regression model, and the SEs were used to calculate the 95 percent confidence interval. In the adjusted model, we controlled for demographic factors such as age, gender, food insecurity, and family members. Although the missing variables were not missing completely at random (MCAR), each item’s missing rate was generally low, under 10%. The proportion of missing data ranged from 0.01% for family size to 7.1% for income loss. Thirty-four with missing data for covariates were not included in multivariable analysis.

### Ethical permission

Verbal consents were taken for the study as they expressed conviction in signing or giving fingerprints on any paper, and also, most of them were analphabetic. They were reassured that all the information collected would be kept strictly confidential and would not be used for anything other than research purposes. However, they were provided with a consent paper with detailed contact information of the research investigators for any future query. The Institutional Review Board at North South University, Bangladesh approved the study protocol (2020/OR-NSU/IRB/1003).

## Results

### Response rate

Of the 540 households sampled, 13 were excluded because the head of the household did not consent. We found 8 of the participants did not complete the questionnaire for BSRS-5. An additional 41 households were excluded because no persons were eligible to be included in the sample during the study period. Finally, in our analysis, we had 478 samples, giving an 88.5% response rate. The details of the response rate calculation are given in the **(S2 Appendix in S1 File)**.

### Characteristics of participants

The mean age (standard deviation) of the 478 participants was 37.02 (12.6) years. Among the participants, 169 (35.4%) were female. According to the wealth index score, 401 (86.1%) of the respondents were from the poor category. Also, 107 (24.1%) respondents reported no income or loss of more than 75% of their pre-cyclone income. Moreover, 66 (13.8%) of the respondents reported displacement from the house due to damaged houses following the cyclone.

### Prevalence of household food insecurity

The characteristics of participants by the four categories of household food insecurity are given in **Table 1**. The prevalence of severe household food insecurity was 40.8% following the cyclone Amphan in coastal Bangladesh. The result indicates that many of the households from Amphan survivors often experienced any of the three most severe conditions: ran out of food, went to bed hungry, or went a whole day and night without eating. Furthermore, the prevalence of moderate household food insecurity was 37.0% which indicates 37% of the households from Amphan survivors eat a monotonous diet or undesirable foods sometimes or often, and/or has started to cut back on quantity by reducing the size of meals or number of meals, rarely or sometimes. It appears that moderate and severe food insecurity was higher among females than males (54.4% of the females and 33.3% of the males reported severe food insecurity).

**Table 1:** Characteristics of the participants by household food insecurity.

Variables	Severely food-insecure	Moderately food-insecure	Mildly food-insecure	Food-secure	Total
	(n = 195, 40.8%)	(n = 177, 37.0%)	(n = 78, 16.3%)	(n = 28, 6.0%)	
**Gender (n = 478)**					
Female	92 (54.4%)	58 (34.3%)	14 (8.3%)	5 (3.0%)	169 (35.4%)
Male	104 (33.7%)	117 (37.9%)	65 (21.0%)	23 (7.4%)	309 (64.6%)
**Age-group (n = 476)**					
18–24	26 (37.7%)	26 (37.7%)	13 (18.8%)	4 (5.8%)	69 (14.5%)
25–34	62 (40.3%)	63 (40.9%)	21 (13.6%)	8 (5.2%)	154 (32.4%)
35–44	45 (38.5%)	47 (40.2%)	18 (15.4%)	7 (6.0%)	117 (24.6%)
45–54	35 (40.7%)	27 (31.4%)	19 (22.1%)	5 (5.8%)	86 (18.1%)
≥55	26 (52.0%)	12 (24.0%)	8 (16.0%)	4 (8.0%)	50 (10.5%)
**Marital status (n = 477)**					
Single	17 (29.3%)	24 (41.4%)	15 (25.9%)	2 (3.4%)	58 (12.2%)
Married	166 (41.1%)	148 (36.6%)	64 (15.8%)	26 (6.4%)	404 (84.7%)
Divorced/Separated/ Widowed	13 (86.7%)	2 (13.3%)	0	0	15 (3.1%)
**Household size (n = 478)**					
≤4 members	107 (44.6%)	89 (37.1%)	32 (13.3%)	12 (5.0%)	240 (50.2%)
5–6 members	65 (38.0%)	61 (35.7%)	34 (18.7%)	11 (6.4%)	171 (35.8%)
≥7 members	24 (35.8%)	25 (37.3%)	13 (19.4%)	5 (7.5%)	67 (14.0%)
**Number of children (n = 478)**					
None	43 (37.7%)	41 (36.0%)	21 (18.4%)	9 (7.9%)	114 (23.8%)
1–2	97 (39.4%)	97 (39.4%)	39 (16.1%)	13 (5.1%)	246 (51.5%)
≥3	56 (47.5%)	37 (31.4%)	19 (16.1%)	6 (5.1%)	118 (24.7%)
**Wealth Index (n = 466)**					
Poorest/poorer	178 (44.8%)	151 (37.7%)	60 (15.0%)	12 (3.0%)	401 (86.1%)
Middle	13 (30.2%)	14 (29.2%)	12 (27.9%)	9 (20.9%)	48 (10.3%)
Richer/Richest	0	4 (17.6%)	6 (41.2%)	7 (41.2%)	17 (3.6%)
**Chronic disease status (n = 478)**					
Absent	89 (34.5%)	93 (36.0%)	59 (22.9%)	17 (6.6%)	258 (54.0%)
Present	107 (48.6%)	82 (37.3%)	20 (9.1%)	11 (5.0%)	220 (46.0%)
**Income loss due to Cyclone (n = 444)**					
No loss/≤25% loss	17 (23.0%)	27 (36.5%)	18 (24.3%)	12 (16.2%)	74 (16.7%)
25–75% loss	108 (42.0%)	105 (40.9%)	32 (12.5%)	12 (4.7%)	257 (57.9%)
No income/≥ 75% loss	55 (48.7%)	26 (23.0%)	29 (25.7%)	3 (2.7%)	1113 (25.5%)
**Post-cyclone housing condition (n = 478)**					
Not damaged	14 (17.7%)	39 (49.4%)	13 (16.5%)	13 (16.5%)	79 (16.5%)
Partially damaged	139 (41.7%)	120 (36.3%)	61 (18.3%)	13 (3.9%)	333 (69.7%)
Housing displacement for severe damaged houses	43 (65.2%)	16 (24.2%)	5 (7.6%)	2 (3.0%)	66 (13.8%)

Moreover, the elderly population (55 and above) reported severe food insecurity than the younger people (52% of older adults and about 38% of younger adults complained about severe food insecurity). The prevalence of severe food insecurity was the highest among the poorer or poorest people (44.8%), whereas none of the participants from the wealthier people reported severe food insecurity. The table shows that 13.8% of the households were completely damaged (i.e., unlivable condition) due to the cyclone Amphan and more than 65% of them had severe food insecurity.

### Prevalence of psychological symptoms

**Fig 1:**
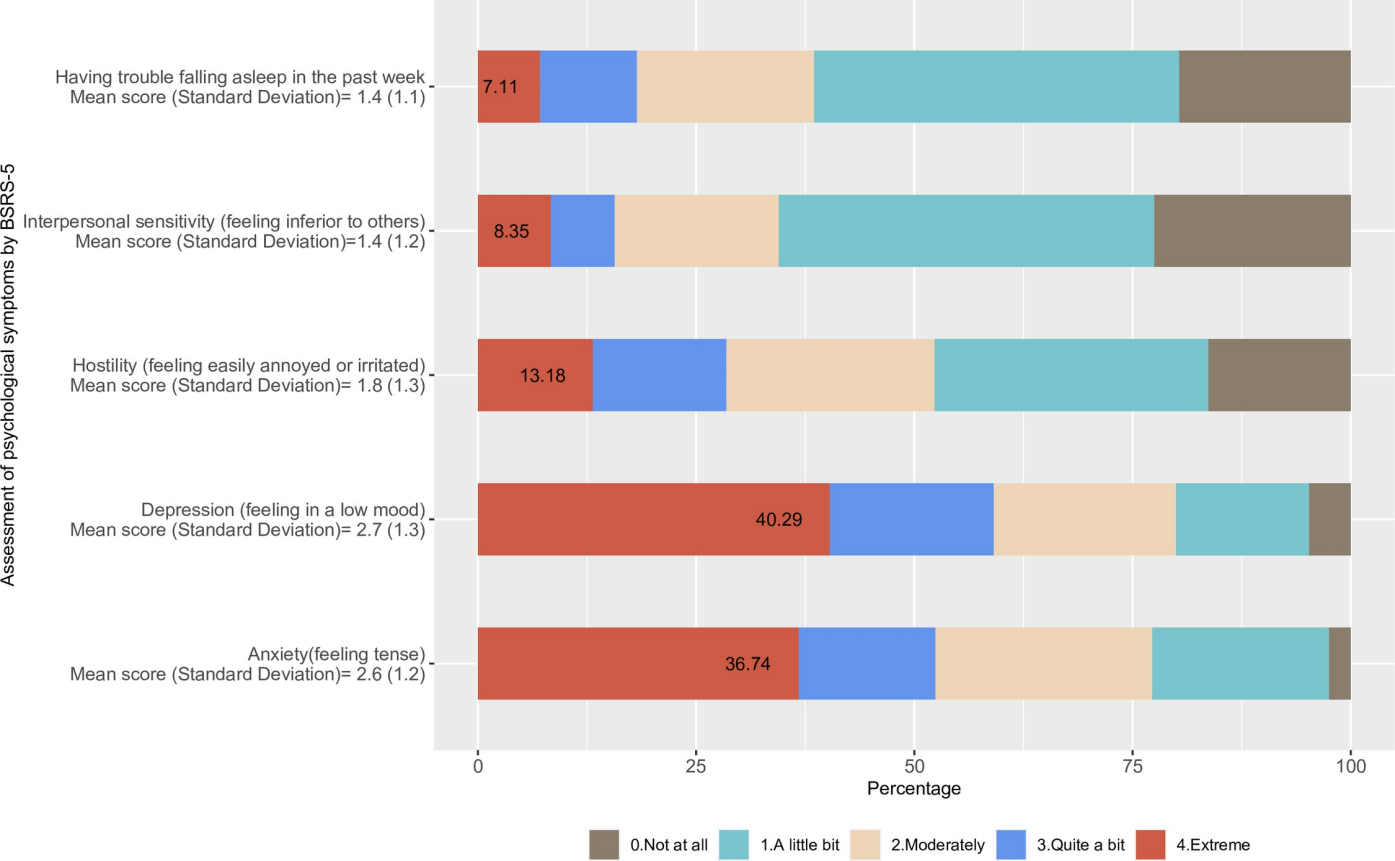
Prevalence of five-item psychological symptoms.

The five-item Brief Symptom Rating Scale (BSRS-5) provides the psychological symptoms shown in **Fig 1**. The figure indicates that psychological symptoms when presenting with depression or anxiety were very common in the community. For example, it appears that 36.7% of the participants reported severe-anxiety, and 40.3% of the participants reported severe-depression. As shown in Table 2, the overall prevalence of moderate-symptoms and severe-symptoms of mental health disorder was 46.5% and 9.2%, respectively.

**Table 2:** Characteristics of the participants by psychological symptoms.

Variables	Normal	Mild mood-disorder	Moderate mood-disorder	Severe mood-disorder	Total
	(n = 84, 17.6%)	(n = 127, 26.6%)	(n = 213, 44.7%)	(n = 53, 11.1%)	
**Gender (n = 477)**					
Female	20 (11.9%)	38 (22.6%)	91 (54.2%)	19 (11.3%)	168 (35.2%)
Male	64 (20.7%)	89 (28.8%)	122 (39.5%)	34 (11.0%)	309 (64.8%)
**Age-group (n = 476)**					
18–24	14 (20.0%)	18 (25.7%)	30 (42.9%)	8 (11.4%)	70 (14.7%)
25–34	32 (20.8%)	40 (26.0%)	64 (41.6%)	18 (11.7%)	154 (32.4%)
35–44	21 (18.1%)	39 (33.6%)	48 (41.4%)	8 (6.9%)	116 (24.4%)
45–54	9 (10.6%)	20 (23.5%)	45 (52.9%)	11 (12.9%)	85 (17.9%)
≥55	7 (14.0%)	9 (18.0%)	26 (52.0%)	8 (16.0%)	50 (10.5%)
**Marital status (n = 477)**					
Single	16 (27.1%)	13 (22.0%)	22 (37.3%)	8 (13.6%)	59 (12.4%)
Married	67 (16.7%)	114 (28.4%)	180 (44.8%)	41 (10.2%)	402 (84.5%)
Divorced/Separated/ Widowed	1 (6.7%)	0 (0.0%)	11 (73.3%)	3 (20.0%)	15 (3.2%)
**Household size (n = 477)**					
≤4 members	40 (16.7%)	56 (23.3%)	115 (47.9%)	29 (12.1%)	240 (50.3%)
5–6 members	28 (16.5%)	49 (28.8%)	78 (45.9%)	15 (8.8%)	170 (35.6%)
≥7 members	16 (23.9%)	22 (32.8%)	20 (29.9%)	9 (13.4%)	67 (14.0%)
**Number of children (n = 477)**					
None	26 (22.8%)	27 (23.7%)	48 (42.1%)	13 (11.4%)	114 (23.9%)
1–2	36 (18.7%)	69 (28.0%)	105 (42.7%)	26 (10.6%)	246 (51.6%)
≥3	12 (10.3%)	31 (26.5%)	60 (51.3%)	14 (12.0%)	117 (24.5%)
**Wealth Index (n = 466)**					
Poorest/poorer	61 (15.2%)	103 (25.8%)	190 (47.5%)	46 (11.5%)	400 (86.1%)
Middle	12 (25.0%)	14 (29.2%)	17 (35.4%)	5 (10.4%)	48 (10.3%)
Richer/Richest	9 (52.9%)	3 (17.6%)	5 (29.4%)	0 (0.0%)	17 (3.6%)
**Chronic disease status (n = 477)**					
Absent	56 (21.7%)	65 (25.2%)	111 (43.0%)	26 (10.1%)	258 (54.1%)
Present	28 (12.8%)	62 (28.3%)	102 (46.6%)	27 (12.3%)	219 (45.9%)
**Income loss due to Cyclone (n = 443)**					
No loss/≤25% loss	24 (32.4%)	17 (23.0%)	29 (39.2%)	4 (5.4%)	74 (16.7%)
25–75% loss	41 (16.0%)	76 (29.7%)	112 (43.8%)	27 (10.5%)	256 (57.8%)
No income/≥ 75% loss	12 (10.6%)	27 (23.9%)	59 (52.2%)	15 (13.3%)	113 (25.5%)
**Post-cyclone housing condition (n = 477)**					
Not damaged	30 (38.5%)	24 (30.8%)	15 (19.2%)	9 (11.5%)	78 (16.4%)
Partially damaged	51 (15.3%)	85 (25.5%)	159 (47.7%)	38 (11.4%)	333 (69.8%)
Completely damaged house and needed renovation for living	3 (4.5%)	18 (27.3%)	39 (59.1%)	6 (9.1%)	66 (13.8%)
**Household food insecurity (n = 476)**					
Food secured	12 (42.9%)	6 (21.4%)	9 (32.1%)	1 (3.6%)	28 (5.9%)
Mild food insecurity	29 (36.7%)	5 (6.3%)	39 (49.4%)	6 (7.6%)	79 (16.6%)
Moderate food insecurity	24 (13.7%)	68 (38.9%)	68 (38.9%)	15 (8.6%)	175 (36.8%)
Severe food insecurity	18 (9.3%)	48 (24.7%)	101 (50.0%)	26 (16.0%)	194 (40.8%)

### Prevalence of suicidal ideation

The prevalence of SI was 10.9%, and the results are given in the **(S2 Table in S1 File)**. The table indicates household food-security plays a significant role in reducing psychological distress with SI. The prevalence of SI in food-secure houses was 5.7%, while the prevalence was 17.6% from households with severe food insecurity. The post-cyclone stressors like income loss and housing conditions also had vital roles on SI. We found that about 14% of the participants reported psychological distress with SI, who did not have an income or had a three-fourth loss of their income due to the cyclone Amphan. In comparison, the number is 6.7% among those who did not lose income or had a slight loss (less than 25% from their pre-cyclone income). Moreover, 13.6% of the participants who reported displacement of houses had psychological symptoms with SI, while it is 3.8% for the participants who did not experience damaged-houses following the cyclone.

### Multivariable analysis: Adjusted prevalence ratio for moderate-to-severe psychological symptoms

We estimated adjusted prevalence ratios (aPRs) using log-linear models adjusted for potential confounders. The DAG figure suggests minimal sufficient adjustment variables are age, gender, income loss, and housing condition after cyclone for estimating the direct effect of household food insecurity on moderate-to-severe symptoms of psychological distress. The results of unadjusted and adjusted prevalence ratios (aPRs) are given in **Table 3**. We fitted three models. The first model was a full model with only the minimum of adjusted variables. The following two models were the reduced models, which did not have household food insecurity and income loss. The reason for a reduced model is that there was a collinearity found between household food security and income loss. The mosaic plot between household food security and income loss is given in the **(S2 Fig in S1 File)**. Moreover, the mosaic plots for investigating relationships between other exposures are given in the **(S2 Fig in S1 File)**.

**Table 3:** Adjusted analysis using robust log-linear models.

Variables	Categories	Unadjusted PR	Model 1	Reduced model 1	Reduced model 2
					Adjusted PR
			aPR (95% CI)	aPR (95% CI)	aPR (95% CI)
**Gender**	Male	Reference			
	Female	**1.30(1.11–1.52)**	1.32 (0.96–1.82)	**1.41 (1.06–1.82)**	**1.30 (1.06–1.59)**
**Age-group**	18–24	Reference		Reference	Reference
	25–34	0.98 (0.76–1.27)	0.73 (0.45–1.18)	0.96 (0.69–1.34)	0.82 (0.63–1.07)
	35–44	0.89(0.69–1.18)	**0.55 (0.32–0.94)**	0.87 (0.61–1.25)	0.74 (0.55–1.00)
	45–54	1.21(0.93–1.58)	0.90 (0.53–1.51)	1.27 (0.89–1.81)	0.96 (0.73–1.26)
	≥55	1**.25(0.94–1.67)**	0.92 (0.51–1.66)	1.33 (0.89–1.97)	1.02 (0.76–1.37)
**Household food insecurity**	Food secured	Reference		Reference	
	Mild food insecurity	1.59(0.94–2.72)	1.25 (0.66–2.35)	1.49 (0.92–2.43)	
	Moderate food insecurity	1.32 (0.79–2.23)	1.04 (0.57–1.92)	1.28 (0.80–2.04)	
	Severe food insecurity	**1.85 (1.11–3.07)**	1.33 (0.73–2.44)	**1.63 (1.01–2.64)**	
**Post-cyclone housing condition**	Not damaged	Reference			
	Partially damaged	**1.92 (1.36–2.71)**	**1.89 (1.18–3.02)**		
	Housing displacement for severe damaged houses	**2.22 (1.53–3.21)**	**1.96 (1.17–3.28)**		
**Income loss due to Cyclone**	No loss/≤25% loss	Reference			Reference
	25–75% loss	1.22 (0.92–1.61)	1.23 (0.85–1.78)		1.13 (0.94–1.37)
	No income/≥ 75% loss	**1.47 (1.10–1.96)**	1.35 (0.90–2.04)		**1.25 (1.02–1.54)**

According to the reduced models 1–2, women were more likely than men to experience mental health symptoms. According to the results, women were 1.41 times more prevalent than men to experience psychological symptoms (aPR = 1.41, 95% CI = 1.06–1.82). Although the prevalence of psychological symptoms increased with age, we did not find the variable as significant. The impact of income loss also shows that no paid jobs or a loss of three-fourth of pre-cyclone income had a significant relationship with the growth of moderate-to-severe psychological symptoms (aPR = 1.25, 95% CI = 1.02–1.54). Severe food insecurity in the households also shows the increased prevalence of mental health symptoms compared to food secured houses (aPR = 1.63, 95% CI = 1.01–2.64). Significant home damage was also associated with growing psychological symptoms (aPR = 1.96, 95% CI = 1.17–3.28).

## Discussions

The devastating nature of disasters and their aftermath may increase household food insecurity, income loss, and psychological distress with suicidal ideation. However, no reports in Bangladesh’s context are available regarding food insecurity, income loss, and mental health associated with cyclones, making it difficult to discuss how these events affect psychological distress with suicidal ideation. This study aimed to identify the prevalence of post-cyclone stressors and mental health symptoms in the coastal Bangladesh population following the Cyclone Amphan and examine associations between post-cyclone stressors and mental health symptoms, including suicidal ideation.

We found a high prevalence of moderate-to-severe mental health symptoms following the Cyclone Amphan in coastal Bangladesh. In another study, five to seven months after the hurricane, moderate to severe mental illness and severe mental illness rates in the metropolitan New Orleans sample were 32.0% and 17.0%, respectively [24]. The World Health Organization conducted community surveys in 21 countries with more than 100,000 individuals and found that the 12-month prevalence of SI was approximately 2% and the lifetime prevalence was 9% [18, 25]. We found psychological distress with SI after cyclone Amphan was 10.9%. The prevalence of psychological distress with SI is higher in this study. The high prevalence of mental health symptoms among low-income survivors of Cyclone Amphan, combined with the lack of health assistance, suggests that there needs immediate and long-term mental health support.

Our results indicate a positive association between severe household food insecurity and moderate-to-severe mental health symptoms. A meta-synthesis of observational research shows an association between food insecurity and symptoms of common mental disorders [26]. A few more studies add to the growing body of evidence that food insecurity is related to psychological distress following a disaster [27–29]. The findings of this research have shown a significant association between household food insecurity and mental health symptoms. These results raise awareness of the extent and magnitude of food insecurity in the sense of post-disaster instability faced in everyday life by disaster survivors. This study’s significant associations are consistent with the hypothesis that a causal effect on suicidal ideation is household food insecurity [13, 30, 31]. In a study conducted in the United States, food insecurity was related to positive difficulties, depression, and sleep deprivation [32]. Another study in a low-income context discovered a relationship between food insecurity and poor mental health [26].

Food insecurity is an ongoing public health issue in Bangladesh [30, 31]. In a study carried out in 2018, the Bangladesh Bureau of Statistics reported that about 12% of the urban poor households went to sleep with empty stomachs [33]. Our study found that the household’s severe food insecurity was about 40%, indicating a high number of hungry people in the coastal region of Bangladesh.

After adjusting for possible confounders, no paid-jobs or significant income loss following the Cyclone Amphan was significantly associated with increased psychological distress symptoms. According to a report conducted after the Great East Japan Earthquake of 2011, long-term unemployment induces severe mental illness [34]. Moreover, we found displacement from the house after the Cyclone Amphan significantly associated with growing mental health symptoms. These findings are consistent with those of a study of Thai survivors of the 2004 tsunami, which showed that those displaced by the tsunami had high mental health symptoms [35, 36].

This study’s findings have significant implications for the planning of psychological care services in the aftermath of a disaster. Long-term recovery and symptom trajectories should be addressed in future studies. Our findings show an increase in mental health problems in the aftermath of the Cyclone, raising concerns about the long-term persistence of mental illness.

### Limitations

Several limitations should be taken into account when interpreting the results. First, our study could not capture the trend of food insecurity and symptoms of psychological distress over time, as we considered a cross-sectional study. Second, we did not review physical and mental health treatment availability and utilization in the post-cyclone Amphan period. This action could be significant because the prevalence of severe mental health disorders among respondents may be mitigated by accessing or using available health services. Similarly, several respondents worried about health conditions, but they were not sure about chronic diseases since they did not visit any health facilities. Consequently, the answer to this question was self-reported from the respondents. The use of the HFIAS scale to measure food insecurity was another limitation of this research, since the scale is a household-level measure and the individual experiences of food insecurity of the participants were likely under-reported. Also, we did not consider other post-cyclone stressors such as the intention of migration to big cities, access to health or education, humanitarian services, and so on. These variables could have confounding effects on the prevalence of psychological distress with SI.

## Conclusions

Our findings highlight the importance of early mental health intervention for individuals in coastal Bangladesh. Economic constraints due to the Cyclone Amphan and income loss have worsened food insecurity and resulted in an increased need for mental health support in the population of coastal Bangladesh. The gender gap in food security is more significant among poorer, elderly, and unemployed women. The link between psychological symptoms, suicidal ideation, and food insecurity provides a considerable position for policymakers and clinicians to minimize psychological distress with SI. Sufficient income and secure employment in the community should be targeted after a disaster. With the interventions to reduce the burden of severe psychological illness among coastal Bangladesh residents, immediate and long-term mental health services are needed.

## Supporting information

S1 File (PDF)
