# Supplementary material for: Household food insecurity, income loss, and symptoms of psychological distress among adults following the Cyclone Amphan in coastal Bangladesh
Source: PLoS One. 2021 Nov 2;16(11):e0259098. doi: 10.1371/journal.pone.0259098 (PMC8562802; doi:10.1371/journal.pone.0259098)
Supplement: S1 File — (PDF) [file pone.0259098.s001.pdf]

Table of Contents

S1 Table. Sample allocation by Upazilas 2

S1 Appendix. Sample size estimation 2

S2 Appendix. Calculation of response rate 3

S1 Fig. Mosaic plots between income loss, household food insecurity and demographic variables 5

S2 Table. Unadjusted prevalence ratio of suicidal ideation by socio-demographic factors 7

S2 Fig. Directed acyclic graph (DAG) to adjust confounders in the associations between exposures and symptoms of psychological distress 5

S1 Table. Sample allocation by Upazilas

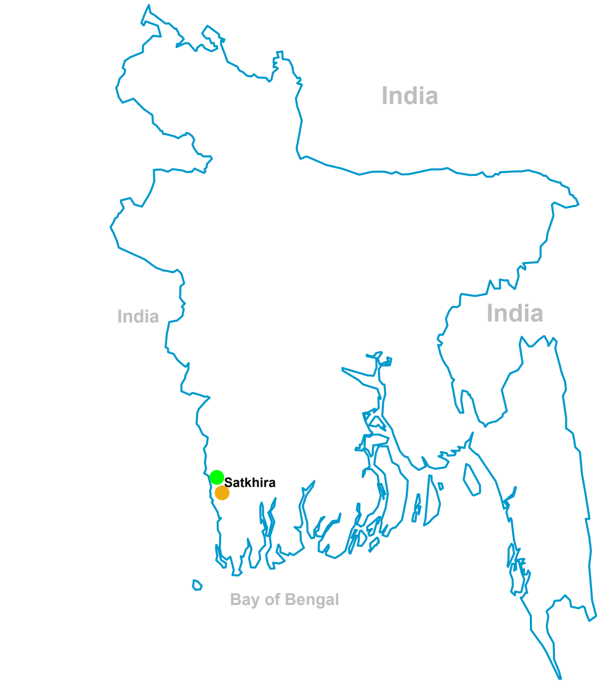

District	Union	Target participants	Respondents
Sathkhira	Shyamnagar	270	250
Sathkhira	Ashashuni	270	238
Total		540	478

Response rate: 88.5%

The Bangladesh map is not copyrighted. The following r code was used to draw the map.

```
library(tidyverse)
library(ggplot2)
library(readr)
library(maps)
library(viridis)
library(maptools)
library(mapdata)
library(RColorBrewer)

map("worldHires", "Bangladesh", col="deepskyblue3",fill=F, lwd=2, mar=c(0,0,0,0))
```

S1 Appendix. Sample size estimation

```
> library(epiR)
> epi.ssclus2estb(b = 270, Py = 0.50, epsilon.r = 0.05 / 0.20, rho = 0.02, nfractional = FALSE, conf.level = 0.95)
$n.psu
[1] 2

$n.ssu
[1] 393

$DEF
[1] 6.38

$rho
[1] 0.02
```

S2 Appendix. Calculation of response rate

The response rate (RR) is the number of complete interviews with reporting units divided by the number of eligible reporting units in the sample. The following formula was used to calculate the response rate [1].

$$\mathbf{RR1} = \frac{\mathbf{I}}{\mathbf{(I + P) + (R + NC + O) + (UH + UO)}}$$

Where, RR1 = Response rate
I = Complete interview
P = Partial interview
R = Refusal and break-off
NC = Non-contact
O = Other
UH = Unknown if household/occupied HU
UO = Unknown, other

The details of these quantities for our study are given in the following:

I and P=The in-person household survey was conducted in which housing units are sampled from an address-based sampling frame of eight selected camps using systematic sampling technique. We consider less than 50% of all applicable questions answered (with other than a refusal or no answer) equals break-off, 50%-80% equals partial, and more that 80% equals complete. We found complete answered from 478 participants (i.e., I=478. We found 8 of the participants did not complete the questionnaire for BRS-5 (i.e., P=8).

R= Refusals and break-offs consist of cases in which some contact has been made with the housing unit and a responsible household member has declined to do the interview, or an initiated interview results in a terminal break-off. Thirteen of the participants refused to take part in the interview, but no one took a terminal break-off after initiating an interview (i.e., R=13).

NC= Non-contacts in in-person household surveys consist of three types: a) unable to gain access to the building, b) no one reached at housing unit, and c) respondent away or unavailable. 6 housings were not accessible due to security condition, and we were not able to reach at housing unit during the interview period (i.e., NC=6).

O= Other cases represent instances in which the respondent is/was eligible and did not refuse the interview, but no interview is obtainable because of: a) death; b) the respondent is physically and/or mentally unable to do an interview; c) language problems; and d) miscellaneous other reasons. We did not face any language problem to exclude participants. We found 2 physically ill (bed-bound or palliative care) patients from household during the interview (i.e., O=2).

UH= Cases of unknown eligibility and no interview include situations in which it is not known if an eligible housing unit exists and those in which a housing unit exists, but it is unknown whether an eligible respondent is present in the housing unit. It was not a scenario in our situation (i.e., UH=0).

UO= Not eligible cases for in-person household surveys include: a) out-of-sample housing units; b) not-a-housing unit; c) vacant housing units; d) housing units with no eligible respondent; and e) situations in which quotas have been filled. In a systematic sampling technique, we found a total of 33 households without any adult respondents during the interview (UO=33).

Thus, the response rate is, RR=478/ (478+8+13+6+2+0+33) = 88.5%

Reference:

[1] The American Association for Public Opinion Research. 2016. *Standard Definitions: Final Dispositions of Case Codes and Outcome Rates for Surveys*. *9th edition*. AAPOR.

S2 Fig. Directed acyclic graph (DAG) to adjust confounders in the associations between exposures and symptoms of psychological distress

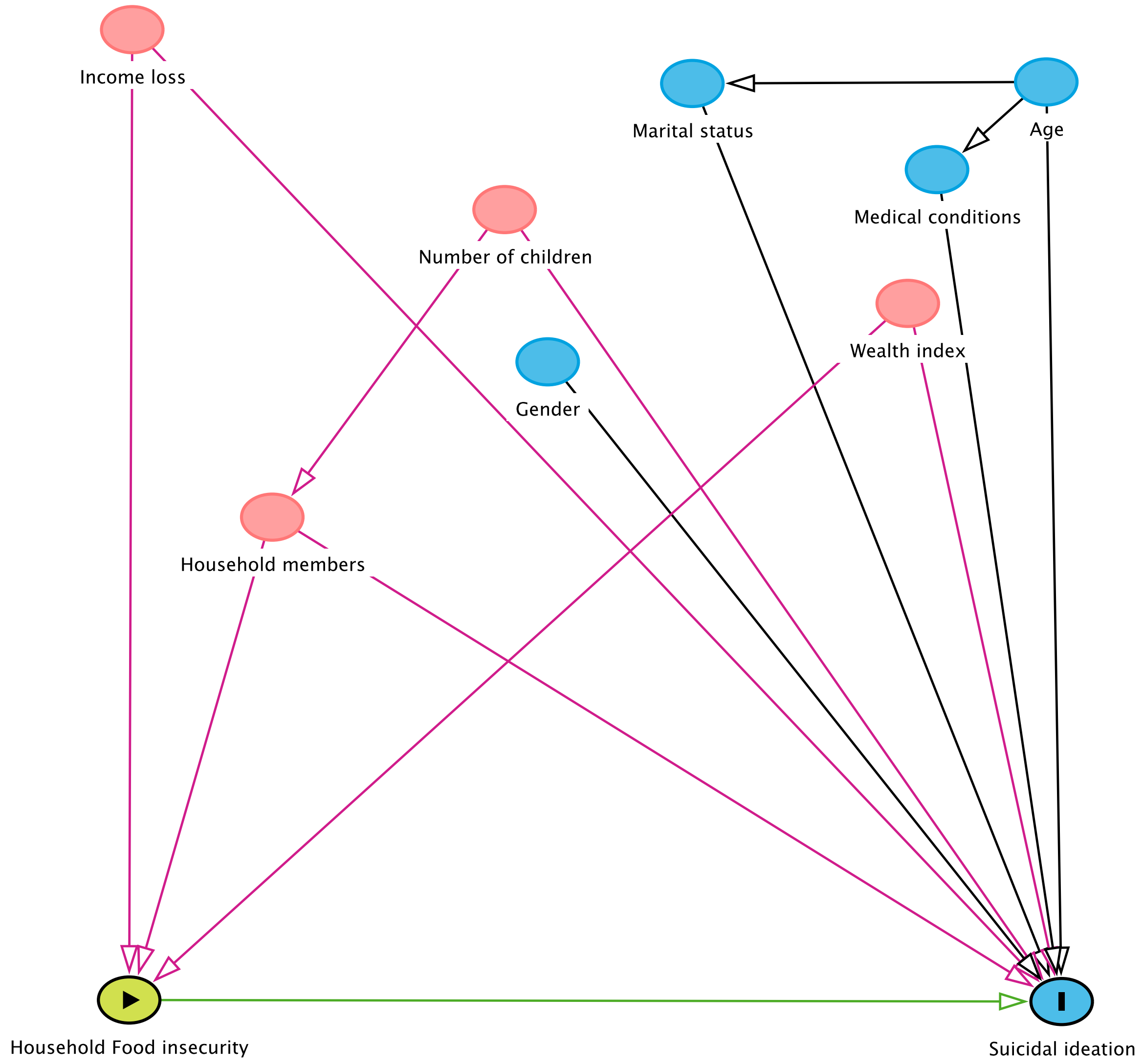

S1 Fig. Mosaic plots between income loss, household food insecurity and demographic variables

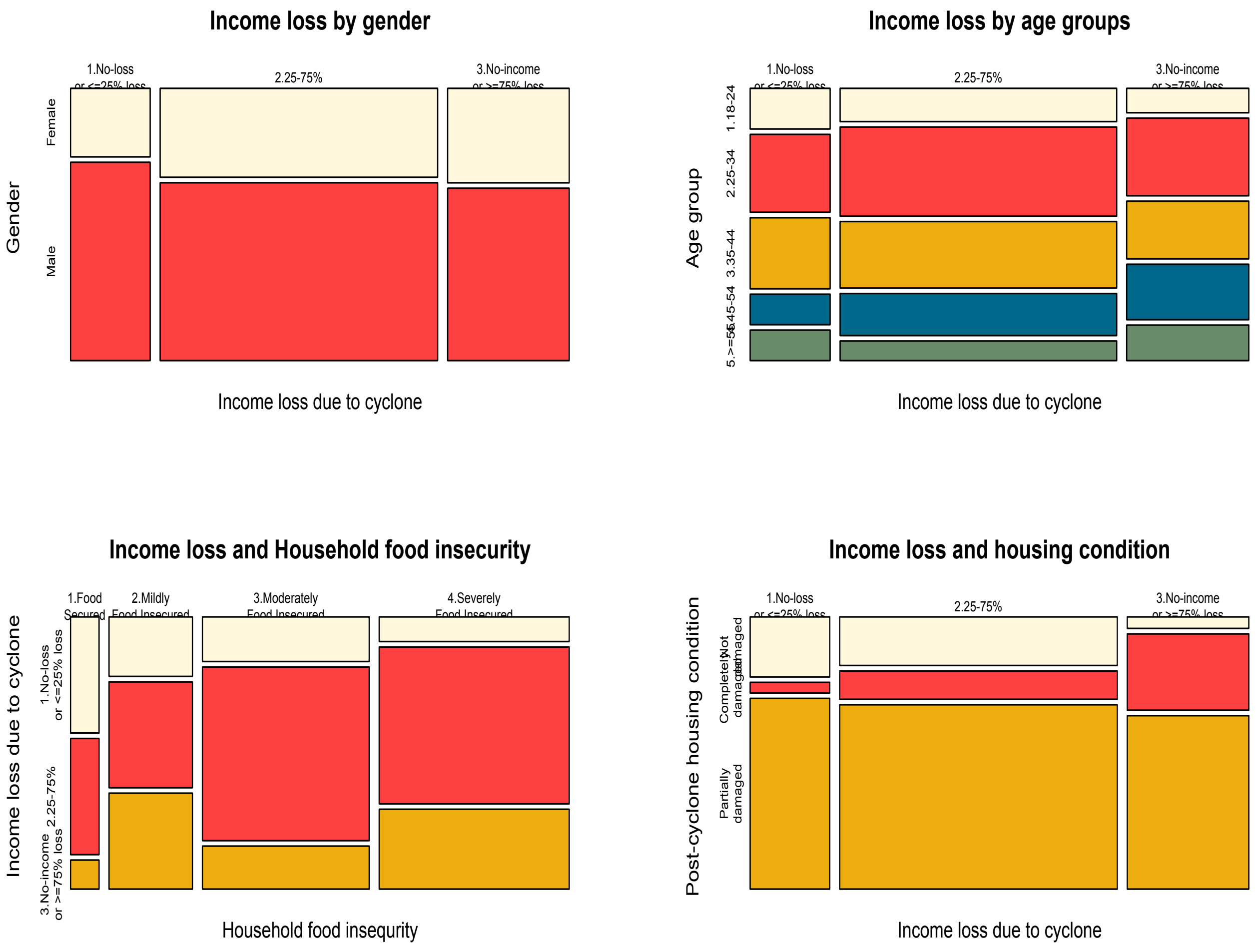

S2 Table. Unadjusted prevalence ratio of suicidal ideation by socio-demographic factors

Variables	Categories	Suicidal ideation		Unadjusted PR (95% CI)	p-Value
		**No (n=425, 89.1%)**	**Yes (n=52, 10.9%)**		
Gender	Female	150 (89.3%)	18 (10.7%)	Reference	
	Male	275 (89.0%)	34 (11.0%)	1.02 (0.60-1.76)	1.00
Age (years)	18-24	61 (87.1%)	9 (12.9%)	Reference	
	25-34	134 (87.0%)	20 (12.4%)	1.01 (0.48-2.10)	1.00
	35-44	110 (94.8%)	6 (6.8%)	0.48 (0.21-1.11)	0.29
	45-54	74 (87.1%)	11 (12.9%)	1.00 (0.44-2.29)	1.00
	≥55	44 (88.0%)	6 (12.0%)	0.93 (0.35-2.45)	1.00
Marital Status	Single	50 (84.7%)	9 (15.3%)	Reference	
	Married	363 (90.3%)	39 (9.7%)	0.63 (0.32-1.24)	0.24
	Divorced/ Separated/ Widowed	12 (80.0%)	3 (20.0%)	1.31 (0.40-4.25)	0.69
Household Members	≤4 members	205 (85.4%)	35 (14.6%)	Reference	
	5-6 members	158 (92.9%)	12 (7.1%)	**0.48 (0.25-0.90)**	**0.02**
	≥7 members	62 (92.5%)	5 (7.5%)	0.51 (0.20-1.25)	0.15
Number of children	None	97 (85.1%)	17 (14.9%)	Reference	
	1-2	221 (89.8%)	25 (10.2%)	0.68 (0.38-1.21)	0.22
	≥3	107 (91.5%)	10 (8.5%)	0.57 (0.28-1.19)	0.15
Wealth Index	Poorer/Poorest	352 (88.0%)	48 (12.0%)	Reference	
	Middle	44 (91.7%)	4 (8.3%)	0.69 (0.26-1.84)	0.63
	Richer/richest	17 (100.0%)	0	-	
Household food-insecurity	Food-secured	28 (100%)	0 (0%)		
	Mild food-insecure	71 (89.8%)	8 (10.1%)		
	Moderate food insecure	165 (94.3%)	10 (5.7%)		
	Severe food-insecure	160 (82.5%)	34 (17.5%)		
Chronic Disease status	Absent	226 (87.6%)	32 (12.4%)	Reference	
	Present	199 (90.9%)	20 (9.1%)	0.73 (0.43-1.24)	0.30
Income loss due to Cyclone	No loss/≤25% loss	68 (91.9%)	6 (8.1%)	Reference	
	25-75% loss	238 (93.0%)	18 (7.0%)	.39 (0.55-2.10)	0.79
	No income/≥ 75% loss	92 (81.4%)	21 (18.6%)	2.29 (0.97-5.41)	0.05
Post-cyclone housing condition	Not damaged	75 (96.2%)	3 (3.8%)	Reference	
	Partially damaged	293 (88.0%)	40 (12.0%)	3.12 (0.99-9.83)	**0.06**
	Completely damaged and lost home	57 (86.4%)	9 (13.6%)	3.54 (1.00-12.51)	**0.04**
